# Housing safety and health academic and public opinion mining from 1945 to 2021: PRISMA, cluster analysis, and natural language processing approaches

**DOI:** 10.3389/fpubh.2022.902576

**Published:** 2022-08-26

**Authors:** Na Li, Rita Yi Man Li, Qi Yao, Lingxi Song, Jirawan Deeprasert

**Affiliations:** ^1^Rattanakosin International College of Creative Entrepreneurship, Rajamangala University of Technology Rattanakosin, Bangkok, Thailand; ^2^College of Computer Science and Information Engineering, Qilu Institute of Technology, Jinan, China; ^3^Sustainable Real Estate Research Center, Department of Economics and Finance, Hong Kong Shue Yan University, Hong Kong, Hong Kong SAR, China; ^4^School of Literature and Journalism, Chongqing Technology and Business University, Chongqing, China; ^5^Chakrabongse Bhuvanarth International Institute for Interdisciplinary Studies, Rajamangala University of Technology Tawan-Ok, Bangkok, Thailand

**Keywords:** safety and health, natural language processing, social media, cluster analysis, PRISMA

## Abstract

Housing safety and health problems threaten owners' and occupiers' safety and health. Nevertheless, there is no systematic review on this topic to the best of our knowledge. This study compared the academic and public opinions on housing safety and health and reviewed 982 research articles and 3,173 author works on housing safety and health published in the Web of Science Core Collection. PRISMA was used to filter the data, and natural language processing (NLP) was used to analyze emotions of the abstracts. Only 16 housing safety and health articles existed worldwide before 1998 but increased afterward. U.S. scholars published most research articles (30.76%). All top 10 most productive countries were developed countries, except China, which ranked second (16.01%). Only 25.9% of institutions have inter-institutional cooperation, and collaborators from the same institution produce most work. This study found that most abstracts were positive (n = 521), but abstracts with negative emotions attracted more citations. Despite many industries moving toward AI, housing safety and health research are exceptions as per articles published and Tweets. On the other hand, this study reviewed 8,257 Tweets to compare the focus of the public to academia. There were substantially more housing/residential safety (n = 8198) Tweets than housing health Tweets (n = 59), which is the opposite of academic research. Most Tweets about housing/residential safety were from the United Kingdom or Canada, while housing health hazards were from India. The main concern about housing safety per Twitter includes finance, people, and threats to housing safety. By contrast, people mainly concerned about costs of housing health issues, COVID, and air quality. In addition, most housing safety Tweets were neutral but positive dominated residential safety and health Tweets.

## Introduction

Housing provides shelter for humans. The United Nations regard housing as a fundamental human right: people enjoy basic accommodation and the right to “adequate housing.” It includes legal security of tenure; availability of services, materials, and infrastructure; affordability; livability; accessibility; location; and cultural suitability (1). As the service life of housing increases, building structures, components, and equipment age gradually, and safety management becomes more complicated, threatening the personal and property safety of owners and users. Thus, there is a need for research that studies safety management for the existing buildings.

Researchers developed different conceptual models to connect various housing aspects (2), including safety and others, affecting people's health in recent decades (3). Some shed light on safety perception and disaster (4, 5), housing safety net (6), public administration (7–9), life environment (10), housing health, etc. Despite the significant differences between the academic research and the public opinions per previous research, none of the articles highlighted this topic to our knowledge, nor social media content analysis. This research attempts to fill this research gap, and the results contribute to academic research on housing safety and public health and provide a reference for future research directions in housing safety and health. A comparison between the two allows us to understand the differences better and offers a hint to design more impactful research in future. The results can also be generalized to other research areas.

This study reviewed housing safety from 1945 to 2021 and addressed the research changes over time, an international collaboration between authors, most productive universities, authors' affiliation, and research focus. It used bibliometrics to study the research focuses, reveal future research direction (11), and help readers understand the research on housing safety in various countries and the primary concerns. It will answer the research questions regarding whether housing safety and health research has attracted more attention in economically developed countries and regions. This study also utilized sentiment analysis methods to study the emotions of Twitter and academic writers. After that, we compare the focus of public opinion expressed on Twitter to academic results.

The research is structured as follows: the second section is the literature review. It reviews housing safety from different perspectives, social media, Twitter, and the differences between academia and the public. Section Research method data source details the research method, including PRISMA, cluster analysis in bibliometrics, and sentiment analysis in natural language processing. Section Results and discussion records the results of academic research on housing safety from five aspects, while section Twitter analysis states the public opinion mining results on Twitter. Most residential and housing Tweets came from Canada and the United Kingdom, respectively, while housing health hazards were from India. Finally, Conclusion concludes the study.

## Literature review

### Housing safety

Rapid urbanization and industrialization have degraded the ecological environment. It has significantly impacted people's lives and property safety (12). With an increase in income and education, people's requirements for living safety continue to increase, and people prefer to live in comfortable homes that are convenient and far from dangerous facilities. Ban and Chen (13) stated that many housing aging problems existed in Shanghai due to rapid urbanization. They investigated housing safety awareness in Shanghai and confirmed the safety status of the premises, asymmetric information, differences among housing users impact housing safety awareness. Husin et al. (14) investigated 24 low-cost houses' safety performance in Kuala Lumpur and residents' satisfaction. The results showed a significant relationship between safety performance and residents' satisfaction.

In recent years, disasters frequently occurred worldwide. Large-scale temporary housing was built after the disaster (15). As the temporary accommodation might be used for a few years, it is significant to ensure temporary housing safety. Among them, site safety and fire risk are the core issues of the safety performance of temporary houses. Hui (16) analyzed location safety and temporary dwellings' fire risk from a safety perspective. The post-disaster fire risk assessment model of temporary houses was established based on the site safety model.

Furthermore, to ensure the safe construction of temporary housing after the disaster, Hassanain et al. (17) proposed a fire safety ranking system (SH-FSRS) for student housing facilities and introduced the developed SH-FSRS through case studies, and suggested improving fire safety performance in these facilities. Dzolev et al. (18) studied the influence of fire load density on fire development with computer code. The fire load density of 120 three-bedroom family apartments in Novi Sad in Serbia was studied. According to their survey results, the fire load density of residential buildings and apartments in Serbia has doubled over the past 30 years. Fire safety has been affected due to an increase in energy released in the burning process. Due to various reasons like natural disasters hazards (4, 19), strengthening housing safety management and improving safety management and maintenance become essential (20).

Many housing safety studies mainly focused on technologies and risk management of housing safety (21). Yu and Fang (22) combined the risk management theory with building safety management, identifying and evaluating risks in buildings' life cycles and putting forward measures for various risk factors. In recent years, risk assessment has been applied in urbanization management including urban flood risk assessment (23), hydrological risk management (24), underground engineering safety risk assessment (25), and landslide challenge assessment during housing development (26); there are few studies on social risk management, especially residents' behavior. Shan et al. (27) put forward the risk management strategy of urban–rural conflict in urbanization in China. Yu et al. (28) developed a model to manage social risks in urban redevelopment projects demolition and investigated the relationship between social risks and stakeholders. A series of studies on residents' behavior based on a questionnaire survey and factor analysis were conducted, such as the influence of residents' collective behavior on housing safety management (29). Previous research on housing safety extends to various topics. Nevertheless, a systematic analysis of housing safety and health research on Twitter is absent, to the best of our knowledge.

### Housing safety and health

Housing safety and health are environmental and social determinants of public health (30). Dunn et al. (31) considered capital construction a feature of research on a healthy social economy. Easterlow and Smith (32) believed that British families attach great importance to safety, linked with housing ownership, and expected to bring practical and psychological benefits. Baker et al. (33) proposed and described a housing insult index (IHI) to capture how housing affects health. There was a hierarchical correlation between housing insults and health in all outcome indicators. In Accra, Ghana, a preliminary study conducted by Arku et al. (34) found that housing conditions, demand, and residents' residence control became significant predictors of self-reported general and mental health status.

Gibson et al. (35) believed that housing and neighborhood conditions were critical social determinants of health. They found that there are three main ways that housing and neighborhood affect health and health inequality: (a) internal housing conditions, (b) regional characteristics, and (c) housing tenure.

For some authors, housing and neighborhood conditions that affect health can be classified into four categories: the unstable home's influence on health (rickety house). Second is the economic burden due to high-cost housing. The third is the impact of family conditions on health (safety and housing quality). Finally, impacts of community on health, including people's living environment and social characteristics (3). In complex housing and health, research methods also advocate continuous innovation.

Lawrence (36) thinks that the interdisciplinary approach highlights the difference between the biomedical model that adopts the explanation of housing and health symptoms and the holistic model that combines biological logic, culture, economy, politics, psychology, and social factors in a new way. Lawrence (37) explained the relevance of interdisciplinary contribution to a more comprehensive understanding of housing density and the complex relationship between housing conditions and health status.

Housing is an essential social determinant of health. Housing experience is significant for the health and wellbeing of vulnerable people such as refugees and asylum seekers. Ziersch et al. (38) interviewed 50 refugees and asylum seekers. The results showed that housing is critical to health and wellbeing. It impacted health through affordability, physical suitability of housing relative to conditions and layout, social aspects such as security and sense of belonging, and security of tenure. Arcury et al. (7) studied the safety, security, hygiene, and privacy of migrant workers' housing using data collected in 183 migrant workers' camps in eastern North Carolina in 2010. They found that migrant workers' housing was insufficient. Overall, 73.8% of the houses were damaged in structure, 52.7% of the homes were unsafe in indoor temperatures, 83.5% of residents feel that their property is unsafe, and 46.2% of the houses have no privacy for bathing or washing (7).

Bamzar (10) studied whether the indoor physical environment of the elderly affected their views on the use of space, falls, and safety. Hirayama (6) discussed the housing safety net system of Japan based on the policy changes. In his study, neoliberalism effectively combined with the traditional low-income housing policy to influence the housing of those at a disadvantage. Venable et al. (4) assessed their views on housing security by investigating more than 450 individuals in communities that received housing reconstruction assistance after Typhoon Yolanda in 2013. They analyzed how housing design factors, post-disaster planning elements, personal characteristics, types of risks, and exposures affected their safety perception. Venable et al. (5) identified families for understanding housing safety. It is suggested that the future post-disaster training plan includes discussing the loading of the housing, focusing on how the components work together, supporting the design and modifying the decision-making to improve housing performance.

### Twitter

As the Internet continues to permeate every aspect of daily life, individuals increasingly share detailed information regarding many aspects of their lives online *via* social media like Twitter (39). As one of the most remarkable social media in the Web 2.0 era (40), Twitter will have 192 million daily active users by 2020. It has been fully integrated into everyday life, despite it sometimes discloses sensitive personal information (41). Apart from its frequent use for personal communication, the usage of Twitter for work is increasing. Twitter citations have been studied as part of the “scientometrics 2.0” project. Twitter substantially affects various fields as knowledge sharing is essential for knowledge management. Bibliometrics and scientometrics have not yet focused their research on Twitter, but the field is increasingly interested in quantifying scholarly activity on the web (42).

Scholars investigated how people used Twitter to share safety knowledge in recent years. For example, Yao et al. (40) analyzed Twitter data *via* content, sentiment, and social network analysis and confirmed Twitter as a valuable tool for sharing relevant knowledge and opinion analyses in construction safety issues. Song et al. (43) analyzed English and French Tweets related to “occupational safety” and investigated languaculture differences' influence on users' behaviors. However, to the best of our knowledge, research on housing safety and health knowledge issue on Twitter is absent.

### Differences between academia and public

Academia has long been perceived as distant from the public and “real” life (44). The “ivory tower” has been used to describe the British academy for several decades and is a metaphor depicting both “outsideness” and the privileged positioning of academics in everyday life (45). Sever et al. (44) explored differences between how people with varying degrees of contact and first-hand experience of academia perceive academics. This had been done by taking three sample groups, those without a university education, students, and academics, across two contexts, Turkey and the United Kingdom, where the academy has a long and well-known history. Thus, two findings could be compared, contrasted, and analyzed: academics' roles and identities. After all, the public and students in both countries believed that academics are irrelevant to their daily life concerns away from the university and inaccessible people hidden in their ivory towers (44).

Ni et al. (46) collected more than 220,000 posts published by approximately 130,000 users regarding the #GeneEditedBabies event. Their results showed that although almost all experts opposed this event, many web-based posts supported it. To understand how self-control strategies helped reach financial goals, Davydenko et al. (47) conducted a meta-analysis to aggregate the latest research on self-control strategies in the financial domain and estimate their overall effectiveness for saving and spending outcomes. Their studies highlighted the academic, online media, and lay-person perspectives. These perspectives overlapped considerably, but there was a gap between what academic study and what people do in their daily lives. There was a more remarkable agreement between the online media and lay perspectives than the academic perspective, suggesting that individuals' personal spending habits followed online media recommendations, rather than academic findings.

## Research method data source

### Bibliometrics analysis

The bibliometric analysis provides an overview of a field of research (48). It studies the research that contributed to a body of knowledge (49), reveals the sources and development of knowledge, and visualizes the connection of knowledge structure in a figure (50). A scientific knowledge map has been a relatively new research method in informatics (50). Cluster analysis classifies elements according to their similarity (51). As cluster analysis efficiently presents the co-citation network based on the article citations and reveals the structure of a particular research field, it is broadly applied for bibliometric research.

Zupic and Cater (52) introduced bibliometrics methods, including citation analysis, co-citation analysis, bibliography coupling, co-author, and co-word analysis, and proposed a bibliometrics research workflow for researchers. Zhou et al. (53) conducted a bibliometric analysis of the articles published in the International Journal of Strategic Property Management from 2008 to 2019 in the Social Science Citation Index database on asset and facility management, property, risk management, residential property value promotion, and housing financing. Moghayedi et al. (54) systematically reviewed the critical success factor for implementing sustainable, innovative, affordable housing. Using a similar approach, Ramos-Rodriguez and Ruiz-Navarro (55) identified research that had the most significant impact on strategic management research and analyzed its changes over time.

Yin et al. (56) discussed the development status of open-source software building information modeling (BIM), revealed the integration between BIM and offsite construction, and determined the research trend based on 4,395 publications about BIM, 2,841 publications about offsite construction, and 113 publications on BIM for offsite construction in Scopus based on bibliometric-qualitative review method. Bibliometrics contributes to a systematic review of the existing studies, providing future research direction. Daim et al. (57) used bibliometrics research and other forecasting methods to forecast fuel cell technology, food technology, food safety technology, and optical storage technology. Using bibliometric analysis, Fahimnia et al. (11) identified the current research areas of interest and potential directions for future research in green supply chain management. They provided a robust roadmap for further analysis. Xia et al. (58) tested the hypothesis that the value of data for scientific investigators, in terms of the impact of the publications based on the data, decreases over time.

The construction of bibliometric maps receives the most attention in the bibliometric literature (59). This research used VOSviewer software (version of 1.6.16) for constructing and viewing bibliometric maps (59). As an essential analytical information visualization tool, VOSviewer can provide a scientific research perspective and accurately mine the research focuses (60). It can display a network of journals, researchers, keywords, and publications based on co-citation, coupling, and co-authorship and obtain visible results (61).

Natural language processing (NLP) was used to analyze the sentiment of the abstract. It provides a clustering function for two terms that appear simultaneously (62). After importing data, the minimum frequency of entries is set. Based on relationship strength and direction, different groups can be found by clustering, network, overlay, and density visualization.

By studying the size of keywords, we know the popularity of the research: significant word size indicates it occurs more frequently. Similarly, the scale of different organizations records their contributions in the field. Similar reasons apply to countries where these publications are published. For example, the structural distribution of research focuses is found through keyword co-occurrence, and the research community is found through author and organization collaboration. The color of an element represents the cluster it belongs to, and different clusters are represented by different colors (62).

Overlay visualization is different from network visualization as the color of overlay visualization has a gradual change effect. Overlay visualization adds a time factor to the visual analysis of objects (62). Different colors correspond to the year when the elements appear (62). Each point in density visualization has a color, indicating the density of elements at that point. By default, the colors range from blue to green to yellow. The more elements near to present, the closer the color to yellow. Works published later are in blue.

### Sentiment analysis

Sentiment analysis is a process of analyzing, inducing, and subjective reasoning texts (40). It is a series of methods, techniques, and tools used to detect the semantics of personal inclination in language. It is the key to a deep semantic understanding of the text (63). Sentiment analysis is the core of text emotion analysis, which refers to the emotional judgment of the text containing personal information. Sentiment can be classified into two categories (positive, negative), three categories (positive, negative, neutral), and multiple categories (happy, excited, sad, angry, etc.) (63). Short-text sentiment analysis is one critical research area in natural language processing. The emotional tendency analysis of the short text can be summarized into text representation, feature extraction, model training, and result analysis (63).

In this study, MeaningCloud was used to investigate emotional content. MeaningCloud is a cloud-based text analysis service. Different APIs provide access to various natural language processing (NLP) tasks, such as automatic classification, sentiment analysis, and topic extraction (43). The API can extract information from unstructured content such as social interactions, articles, and documents (40). In addition, MeaningCloud is more accurate than similar NLP tools (64).

### Data source

Web of Science is a large comprehensive, multidisciplinary, and core journal citation index database. It includes the Science Citation Index (SCI), Social Sciences Citation Index (SSCI), Arts and Humanities Citation Index (A&HCI), Current Chemical Reactions (CCR), Index Chemicus (IC), Science Citation Index Expanded (SCIE), Conference Proceedings Citation Index-Science (CPCI-S), and Conference Proceedings Citation Index-Social Science & Humanities (CPCI-SSH), with ISI Web of Knowledge as the retrieval platform (43).

**Figure 1:**
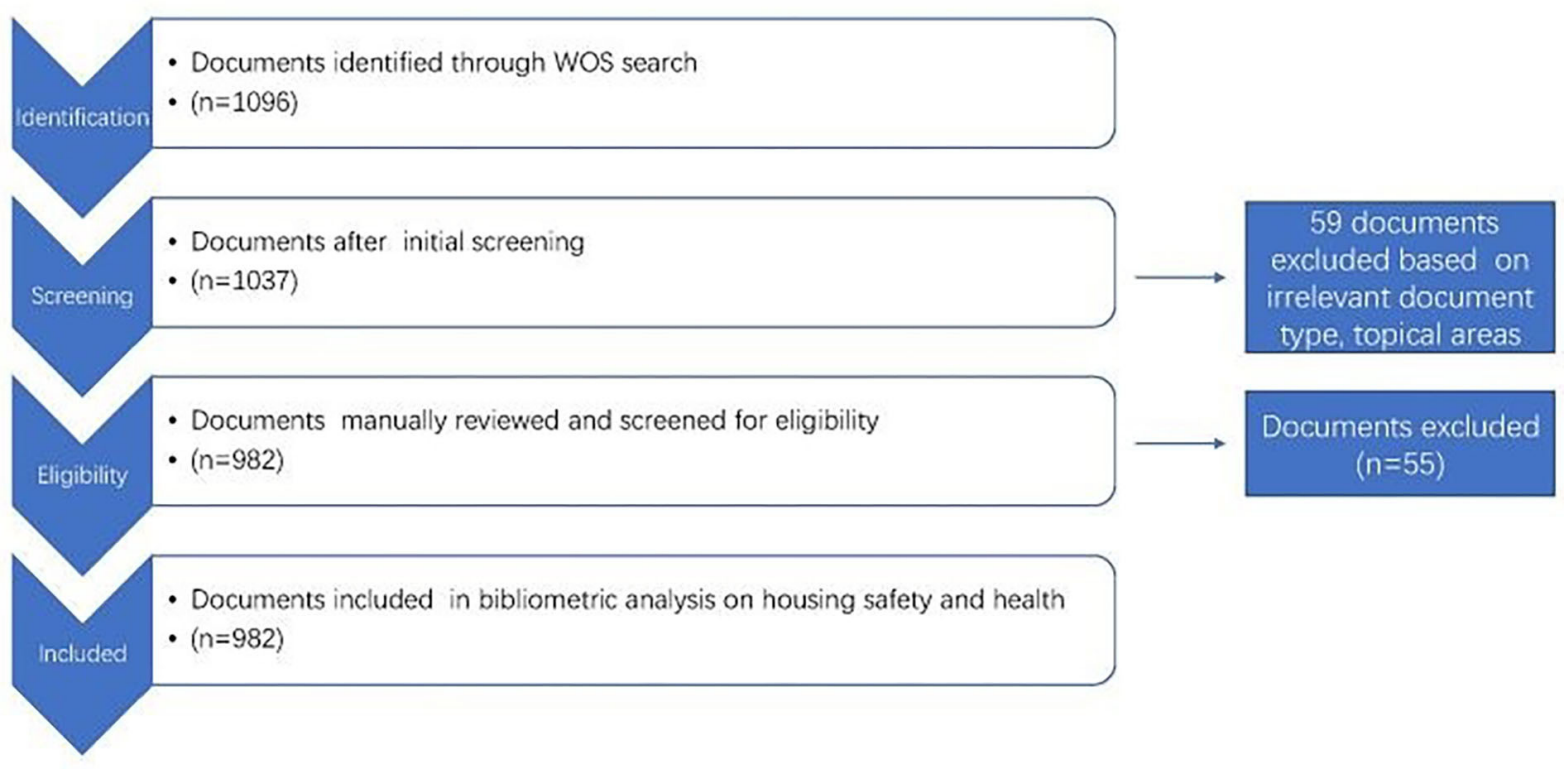
PRISMA for selecting relevant housing safety and health research indexed in WOS.

The Web of Science Core Collection is selected as the data source in this study. According to Preferred Reporting Items for Systematic reviews and Meta-Analyses (PRISMA) data (65), this study extracted original data (65), and 982 results were retained. It followed PRISMA guidelines for conducting systematic research reviews (66). Using keywords “housing safety,” “residential safety,” “intelligent home safety,” “sick house syndrome,” and “housing health hazard” to search for the documents in Web of Science, document details are then imported to Excel to sort, remove duplicate records, and delete documents that do not conform to the research topics and retrieval methods. After the aforementioned treatment, we obtained 1037 pieces of literature. The PRISMA process is presented in Figure 1.

## Results and discussion

### Time development context

**Figure 2:**
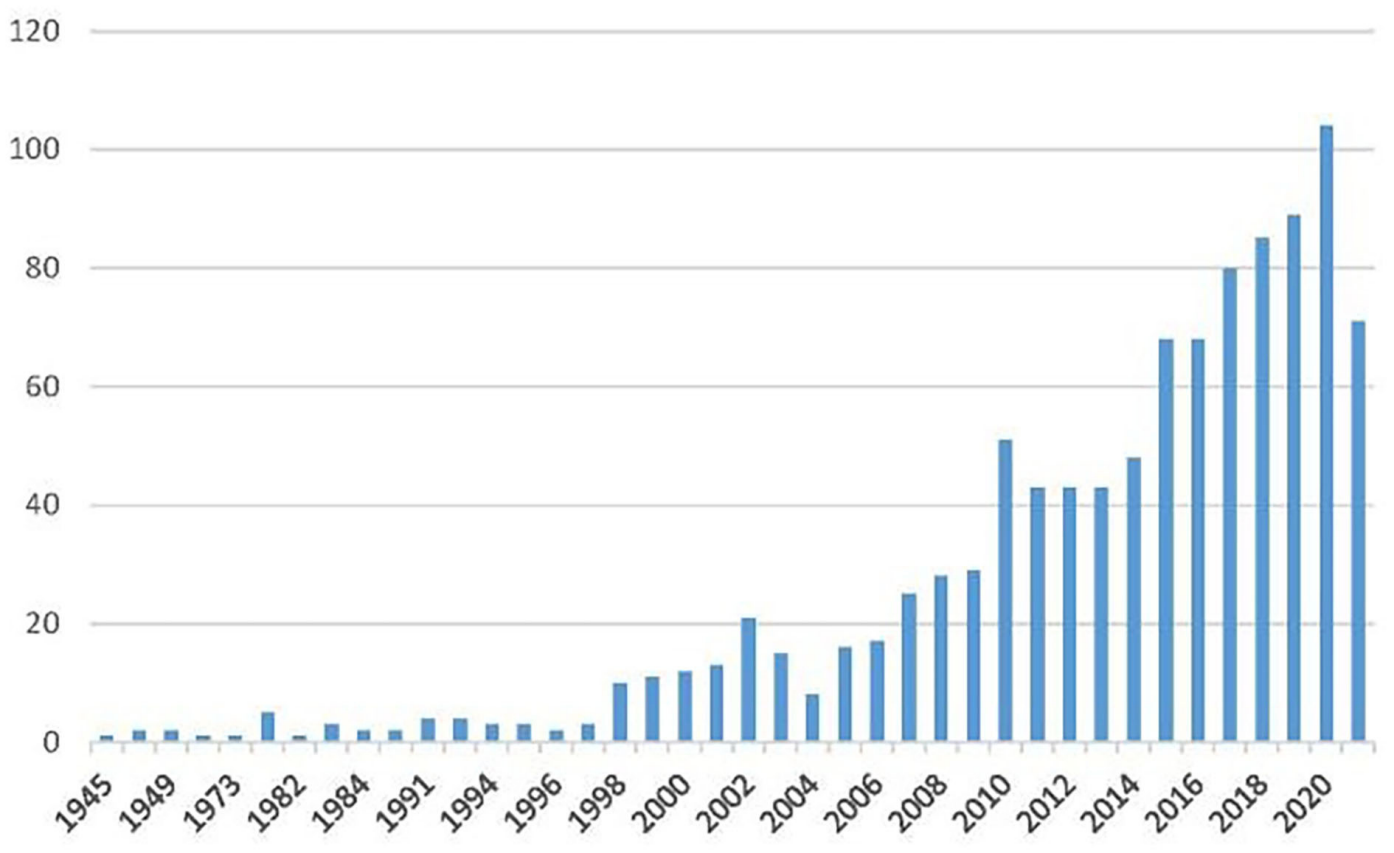
Annual number of published housing safety and health research articles (1945–2021).

The number of articles published each year recorded the trend of publication in the field (Figure 2). It shows an upward trend in three stages. Web of Science covers the articles published since 1900, but we found no related articles from 1900 to 1944. Until 1945, Wilkins published the first article in the British Medical Journal about housing and health. The second stage is from 1945 to 1997. Only 16 published articles were retrieved in this stage, which shows that the research on housing safety is still in the infantry stage. The third stage is from 1998 until now, and the number of published articles accounts for 96% of the total number of articles, which shows housing safety and health research flourished. Since 1998, the number of articles has increased gradually. While the growth rate was relatively slow from 1998 to 2009, published works boomed after 2010.

### International cooperation

**Figure 3:**
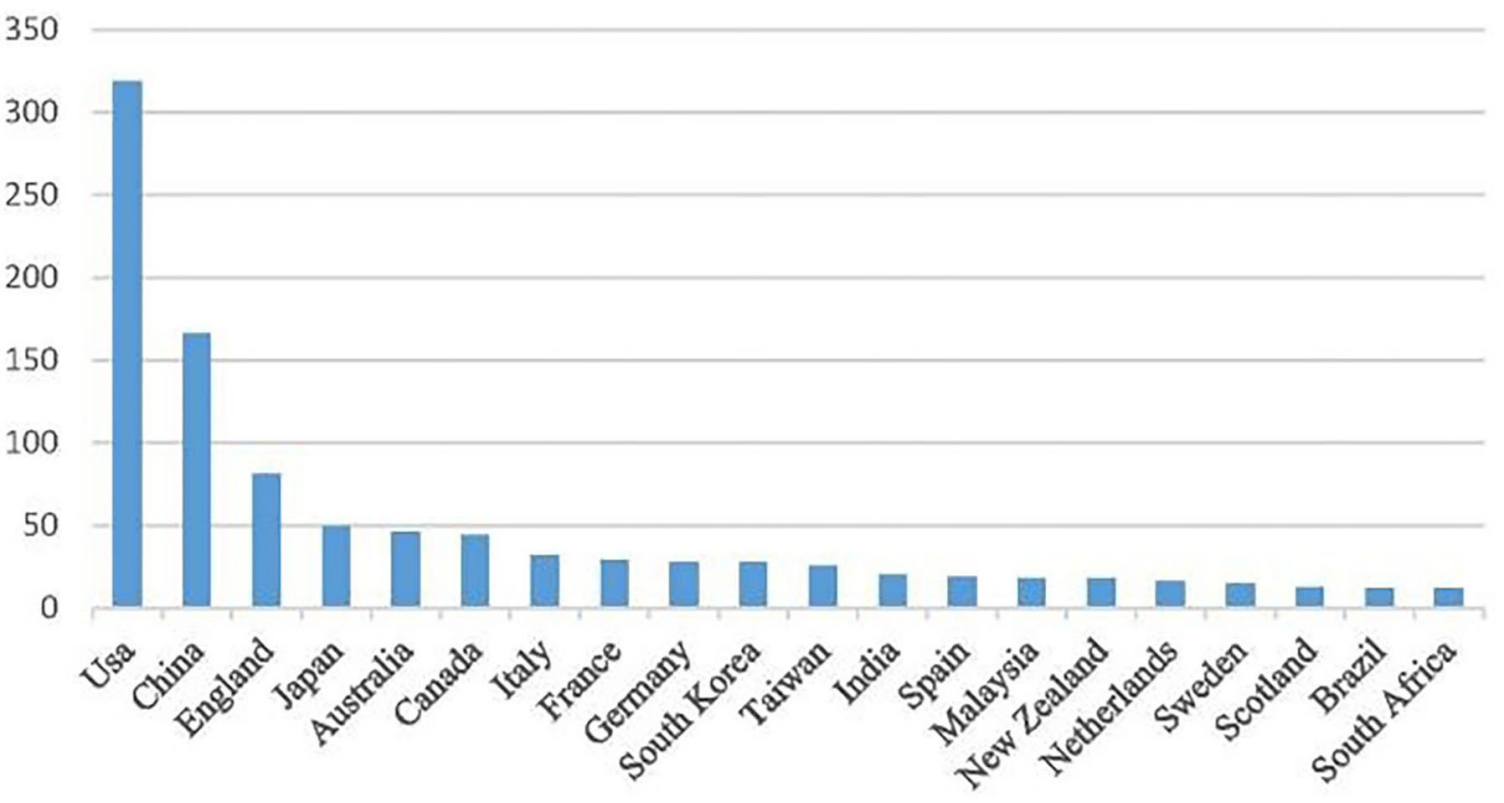
Number of articles published by the top 20 countries.

The top 10 most productive countries are developed countries, except China, which ranked second (Figure 3). The United States has the most significant number of publications (n = 319), accounting for 30.76%. This proves that scholars' housing safety issue in the United States is now a remarkable concern. The number of articles published in China ranked second (n = 166), accounting for 16.01% of published works. Total citations in the United States reached 8,189, and the average cited frequency was 25.67, while the total cited frequency in China was 1,375, with an average citation of 8.28, that is, about one-third of articles written by U.S. authors. Although the number of articles published in China ranked second to the United States, the average number of cited articles is much lower than that in the United States.

On the one hand, due to the late start of real estate-relevant research in China, the number of published articles is not considerable and so is the average citation. In future, Chinese housing safety researchers need to publish articles in top journals and improve the research quality. We speculate that the socialist system may influence housing safety and health issue in China.

**Figure 4:**
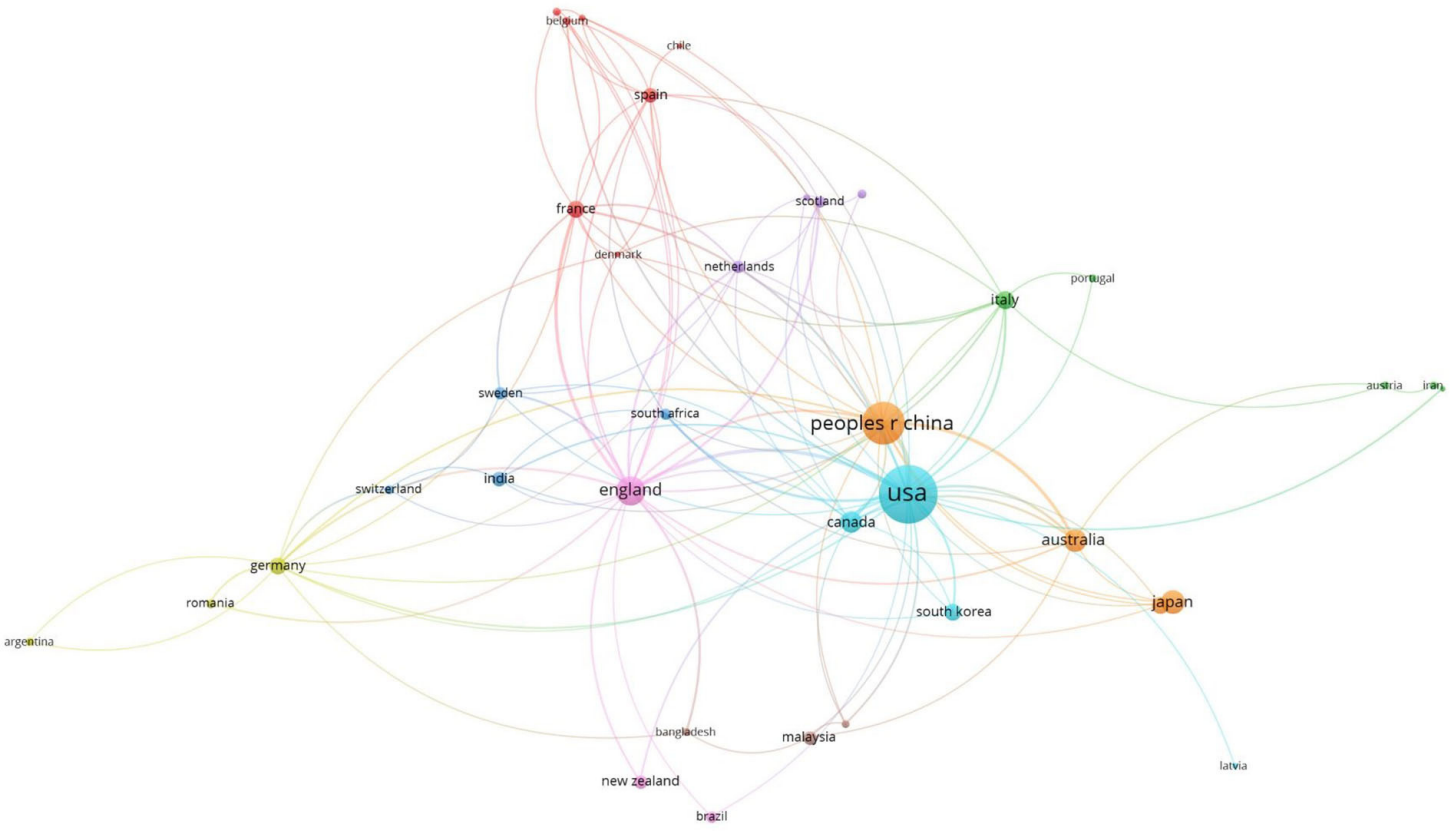
Network visualization analysis of international cooperation of housing safety research.

Figure 4 shows the number of articles, cooperation, and influence among countries. The node size represents the number of articles, and the connection represents co-authorship. The core country represented by the blue cluster is the United States, and its principal research co-authors are from Canada, South Korea, Sweden, and India. China is the core country of the orange cluster, and Australia, Japan, France, and Spain are its main research partners. The core country in the purple cluster is Britain, and the collaborative countries in this cluster include New Zealand, the Netherlands, Scotland, and Brazil. France is the core country shown by the red cluster, and its collaborative countries include Spain and Sweden. The United States is the first country to conduct housing safety research, followed by Japan, but China has shown a significant growth trend in recent years. Although this research started later in China than in the United States, it developed rapidly. We can identify the United States and China as two representative countries. Other countries with apparent element density include Britain, Japan, Australia, and Canada.

### University with the largest number of publications

**Figure 5:**
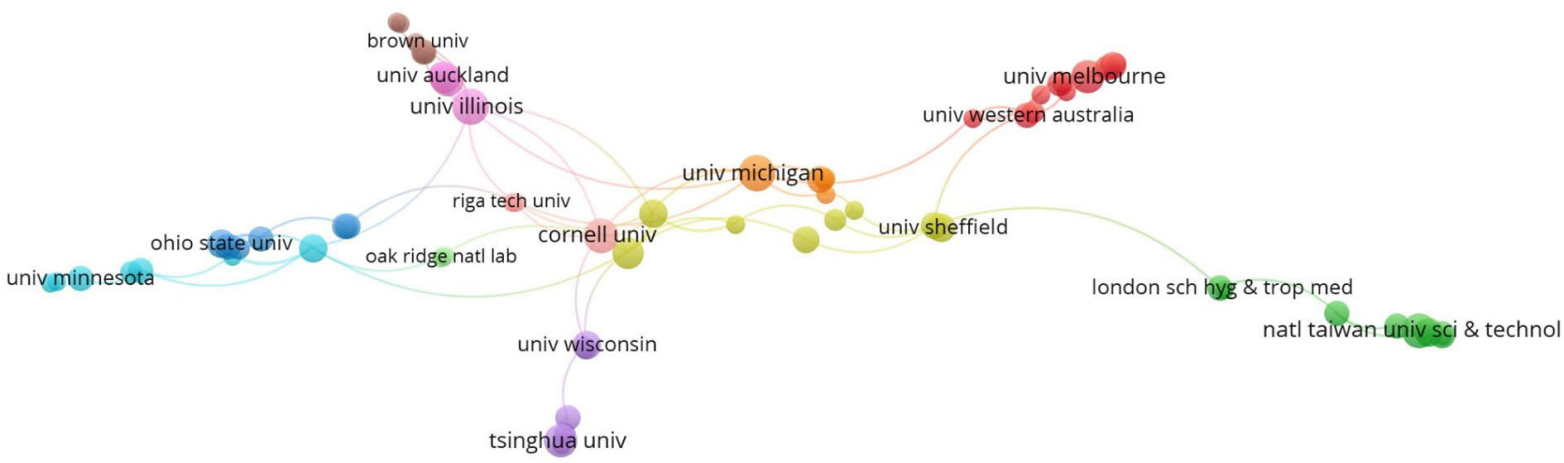
Network visualization analysis of issuing agency of housing safety research.

The University of California recorded the most significant publications (20 articles). The University of London in Britain ranked second (13 articles), the University of Michigan ranked third (12 articles), and Tsinghua University had nine publications, ranking eighth. According to VOSviewer statistics, 1,351 research institutions and 350 institutions jointly published two articles (≥2), accounting for 25.9% of the total cooperative institutions. It shows that inter-institutional cooperation, academic exchanges, and collaboration are not frequent. According to VOSviewer cluster analysis, as shown in Figure 5, there are six small clusters, and the clusters where blue, pink, and orange are located refer to American research institutions. The purple cluster represents Tsinghua University in China as the core. The red group mainly includes the University of Melbourne in Australia; the green cluster shows the close cooperation between the University of London and the National Taiwan University of Science and Technology.

### Authors' collaboration

According to VOSviewer authorship statistics, 3,173 authors have published articles on house safety. After data cleaning, the top 10 authors have been sorted out in Table 1. Table 1 shows that Evans, G.W. of Cornell University, is the author with the most significant number of articles (7), an H-index of 7, and the highest total cited frequency of 1,708 times. The article “The environment of poverty: multiple stress exposure, psychological stress, and social adjustment” published in 2002, was cited 647 times, thus most cited among all authors.

**Table 1:** Top 10 most published researchers on housing safety.

**No**.	**Authors**	**Affiliated institutions**	**Belonging country**	**Number of publications**	**h-index**	**The total cited frequency**
1	Evans, G. W.	Cornell University	USA	7	7	1,708
2	Howden-Chapman, P.	University of Otago	New Zealand	7	4	55
3	Ormandy, D.	University of Warwick	England	6	4	175
4	Arcury, T. A.	Wake Forest University	USA	4	3	106
5	Chan, D. W. M.	Hong Kong Polytechnic University	Hong Kong China	4	3	43
6	Fu, Tat S.	Simpson Gumpertz and Heger Inc.	USA	4	3	28
7	Isidori, D.	AEA SRL	Italy	4	2	17
8	Jacobs, D. E.	Goldblatt Syst LLC	USA	4	4	116
9	Kim, S.	Yonsei University	South Korea	4	2	90
10	Kishi, R.	Hokkaido University	Japan	4	3	261

**Figure 6:**
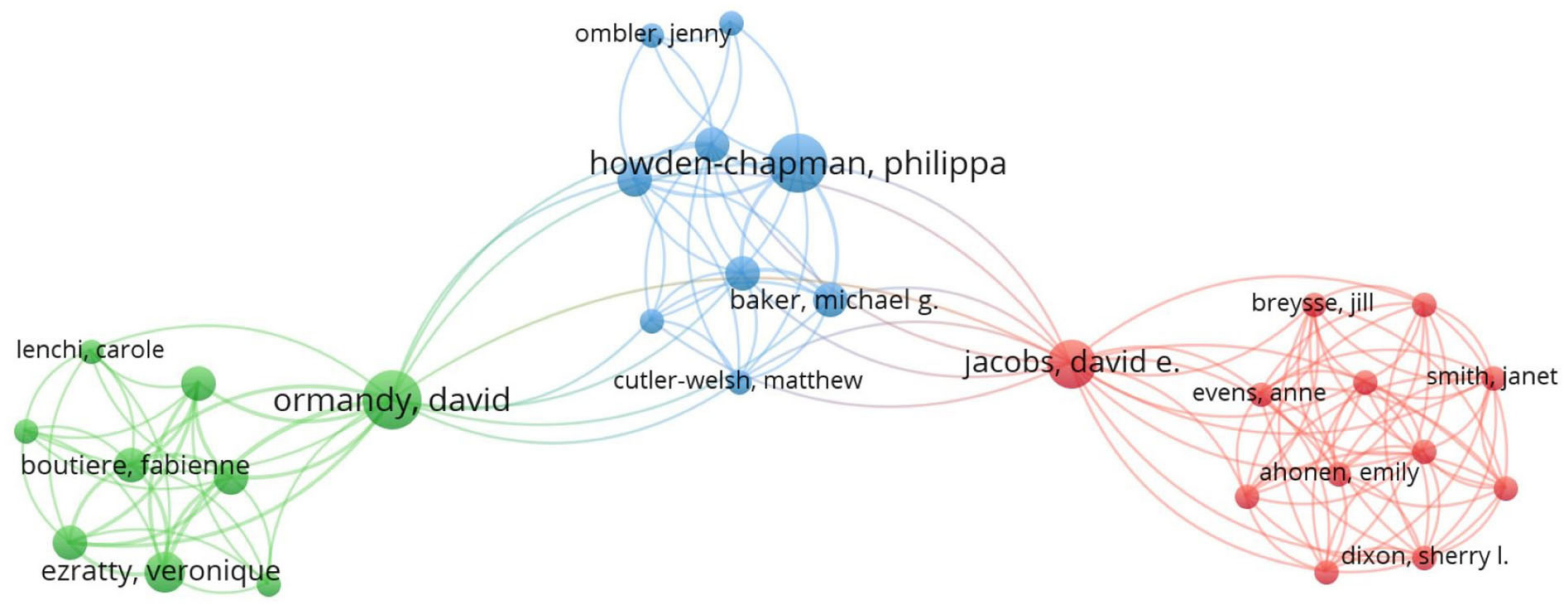
Co-author diagram of housing safety research articles based on VOSviewer.

There are 186 core authors (scholars who have published more than or equal to two articles) in the housing safety study (*N* = 2.802) (67). By analyzing their published articles, we can effectively identify the development context of the field. According to Price's theory, $N\;=\;0.749{\left(N_{\max}\right)}^{\frac{1}{2}}$, there are three clusters. It shows close cooperation within clusters and less cooperation outside clusters. Nmax refers to the most significant number of articles, and N is the minimum number of articles published by core authors. Their cooperation relationship is shown in Figure 6. It reflects that geographical proximity favors cooperation as less cooperation across regions and disciplines.

It can be seen from Figure 6 that three clusters are green cluster with Ormandy, D.; the blue cluster with Howden-Chapman, P.; and the red cluster with Jacobs, D.E. The blue cluster cooperates closely with the green and red clusters, while the green and red clusters have less cooperation.

### Research focuses

#### Most productive journals

The main categories of housing safety research in the Web of Science are engineering (28.9%), public environmental occupancy health (15.1%), environmental sciences and ecology (9.7%), and computer science (8.8%). These four categories account for 63%. By sorting the sources of publications, this study revealed that housing safety is mainly published in Safety Science (impact factor 6.392), followed by the American Journal of Public Health (impact factor 11.561), International Journal of Environmental Research and Public Health (impact factor 4.614), Habitat International (impact factor 5.205), Social Indicators Research (impact factor 2.935), Social Science and Medicine (impact factor 5.379) in 2021 Journal Citation Reports.

#### Bibliometrics and sentiment analysis

**Figure 7:**
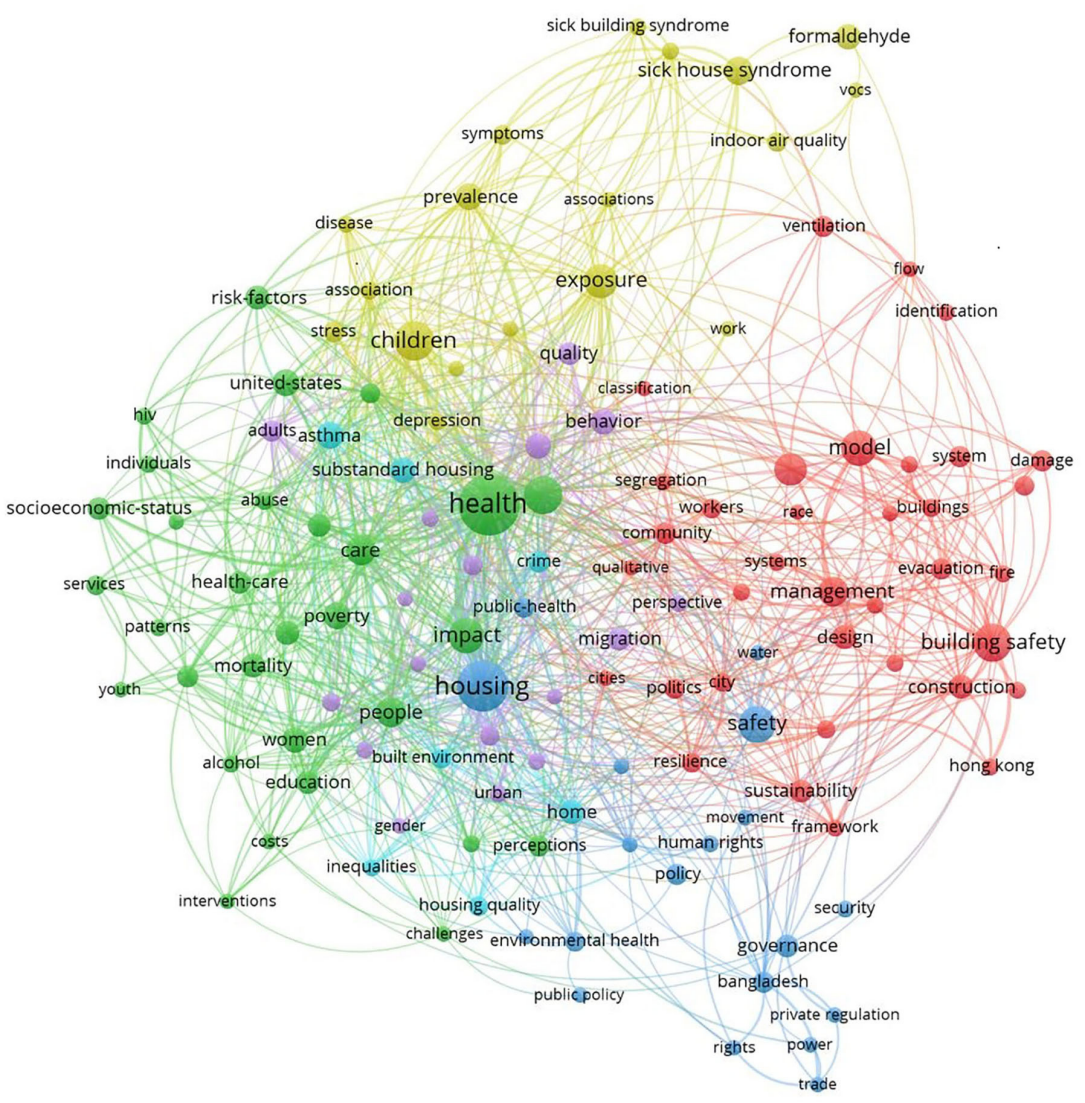
Co-occurrence analysis of keywords.

Using VOSviewer, this study makes a co-occurrence analysis of literature keywords related to housing safety research in the Web of Science Core Collection (Figure 7). It retrieved 1,037 pieces of literature and 127 keywords in six clusters. The higher the frequency of the keyword in the cluster, the larger the sphere in the co-occurrence figure, which also shows that the keyword is a research focus. However, since the same concept can be expressed in different keywords (such as housing safety and residential safety in Figure 7), the sphere size cannot wholly describe the situation. Therefore, a combination of similar keywords is essential for research focus analysis.

The first cluster is the red cluster, which covers safety management-related issues based on 36 keywords, such as building safety, model, performance, management, and design. The second category is health-related green clusters. It covers 30 keywords, including health, risk, impact, and care. The third category is the deep blue cluster with 19 keywords, including housing, safety, government, policy, public health, and rights. The fourth category is the yellow cluster, containing 18 keywords, including children, exposure, formaldehyde, prevalence, sick building syndrome, and sick house syndrome. The fifth category is the purple cluster, which covers 17 keywords, including behavior, environment, migration, and quality. The sixth category is the light blue cluster, with seven keywords: asthma, built environment, crime, home, housing quality, inequalities, and substandard housing. There are more research keywords related to housing health than safety.

Housing safety research in developed countries such as the United States started earlier. Thus, more relevant research is based on various methods and means (68). By contrast, according to national policies and economic development, the number of publications in developing countries is usually less, and there is room for improvement in article quality. However, China is an exceptional case. Although the research on housing safety management in China started later than in the United States, it developed rapidly.

**Figure 8:**
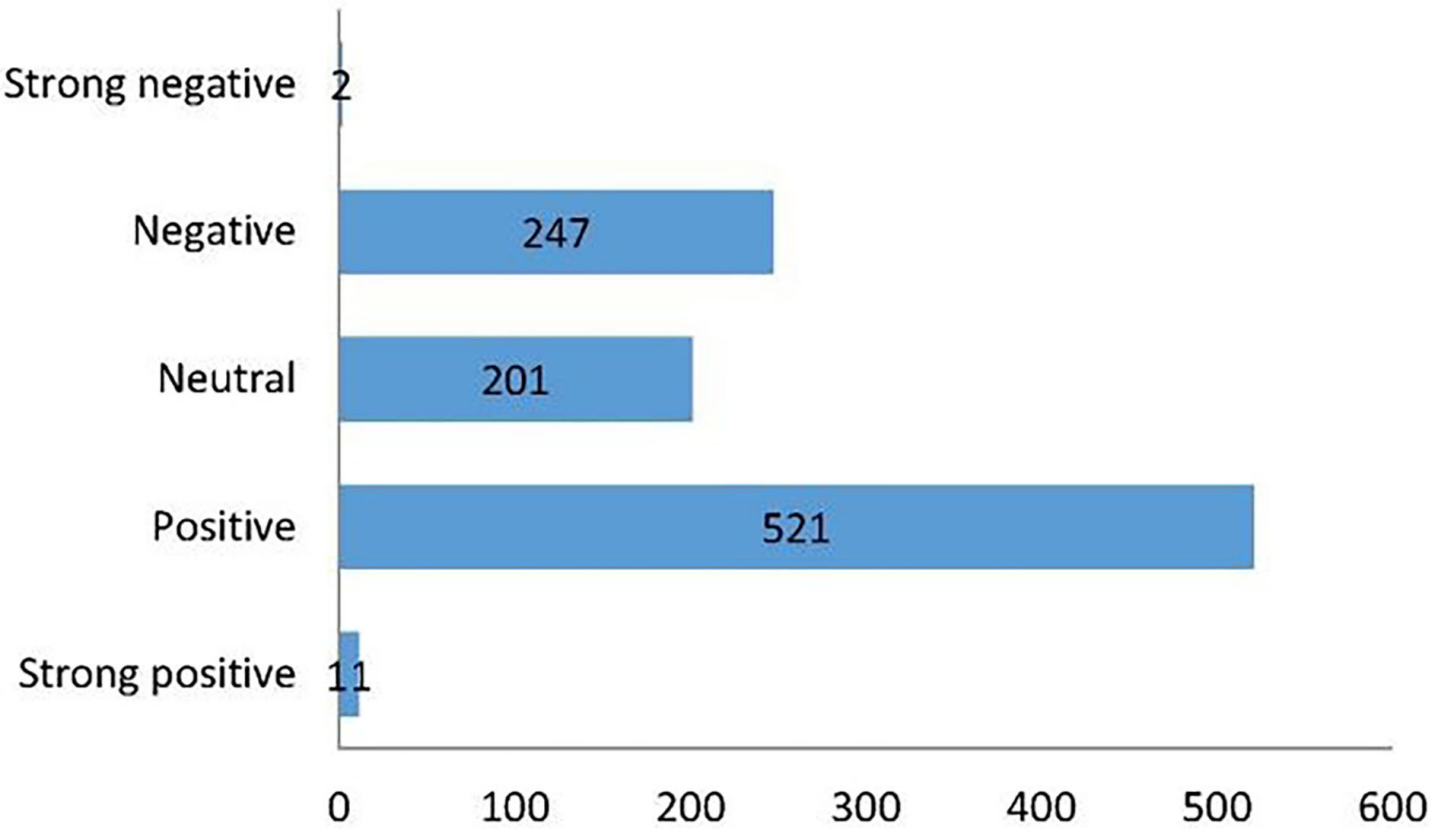
Emotional distribution based on published literature abstracts.

This study analyzed emotions of 982 abstracts. The sentiment distribution is shown in Figure 8. Strong positive, positive, neutral, negative, and strong negative are marked as 2, 1, 0, −1, and −2. Of all the abstracts, the number of strong positives was 11, positive was 521, neutral was 201, negative was 247, and strong negatives was 2, accounting for 1.12, 53.05, 20.47, 25.15, and 0.20%, respectively. The correlation between citations and sentiment is negative (−0.123), indicating that negative abstracts are linked with higher citations, and the average confidence level is 90.48%.

## Twitter analysis

To compare the focus of academia and the public, we studied Tweets in the second part of the research. From 24 January 2022 to 26 January 2022, it used “housing safety,” “residential safety,” “intelligent home safety,” “sick house syndrome,” and “housing health hazard” as keywords and obtained 8,257 results. “Intelligent home safety” and “sick house syndrome” had no result, “housing safety” had 7,513 Tweets, “residential safety” had 685 Tweets, and “housing health hazard” had 59 Tweets. Furthermore, this study analyzed the top opinion leaders for “housing safety,” “residential safety,” and “housing health hazard.” It also conducted a sentimental analysis of Tweets by MeaningCloud.

Regarding housing safety, this research found 7,513 Tweets. According to the in-degree centrality, the top 10 influencers are listed in the following table, out of which, eight were from the United Kingdom, and most came from the government or politics. This shows that the U.K. government's issue of housing safety has been widely concerned. This phenomenon shows that the U.K. government pays the most attention to sharing housing safety issues on Twitter (Table 2).

**Table 2:** Housing safety key opinion leaders in Twitter.

**Twitter ID**	**Description**	**Attribute**	**Place**
Danpriceseattle	CEO just trying to stand up for the underdog	Individual	US
nataliejolyt8n	Big feelings re: EDI, Enviro/Economic Sustainability, Arts. I read all the things. #StAlbert Councilor. Homeland Housing Chair. Volunteer @nilmdtsHQ. She/her.	Councilor	Canada
Michaelgove	MP for Surrey Heath. Secretary of State for Leveling Up, Housing and Communities @luhc	Member of Parliament	UK
Springhousing	Spring Housing Association was set up in 2014 to provide accommodation and support services to individuals who are at risk of homelessness. Charity reg: 1163098	Organization	UK
Zarahsultana	Proudly serving the people of Coventry South as their Labor Member of Parliament 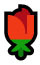 | 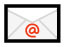 : zarah.sultana.mp@parliament.uk | she/her	Member of Parliament	UK
Carolinelucas	Green MP for Brighton Pavilion, former leader and co-leader of @TheGreenParty, Mum	Member of Parliament	UK
Commonsluhc	We are a cross-party group of MPs in the @HouseofCommons that scrutinizes the work of @luhc. RTs ≠ endorsements.	Political group	UK
team_greenhalgh	Minister for Building Safety, Leasehold and Resilience & Emergencies at @luhc and Fire Minister at @ukhomeoffice - in the Lords	Minister for Building Safety	UK
Lqhomesmatter	Leading charitable housing association and developer. Tweets read Mon-Fri, 9-5. Customer service: @lqcontactus L&Q's response to coronavirus: https://t.co/bZna9zTv07	Organization	UK
Luhc	We are leveling up the UK, regenerating towns and high streets and supporting communities across the country.	Government department	UK

**Figure 9:**
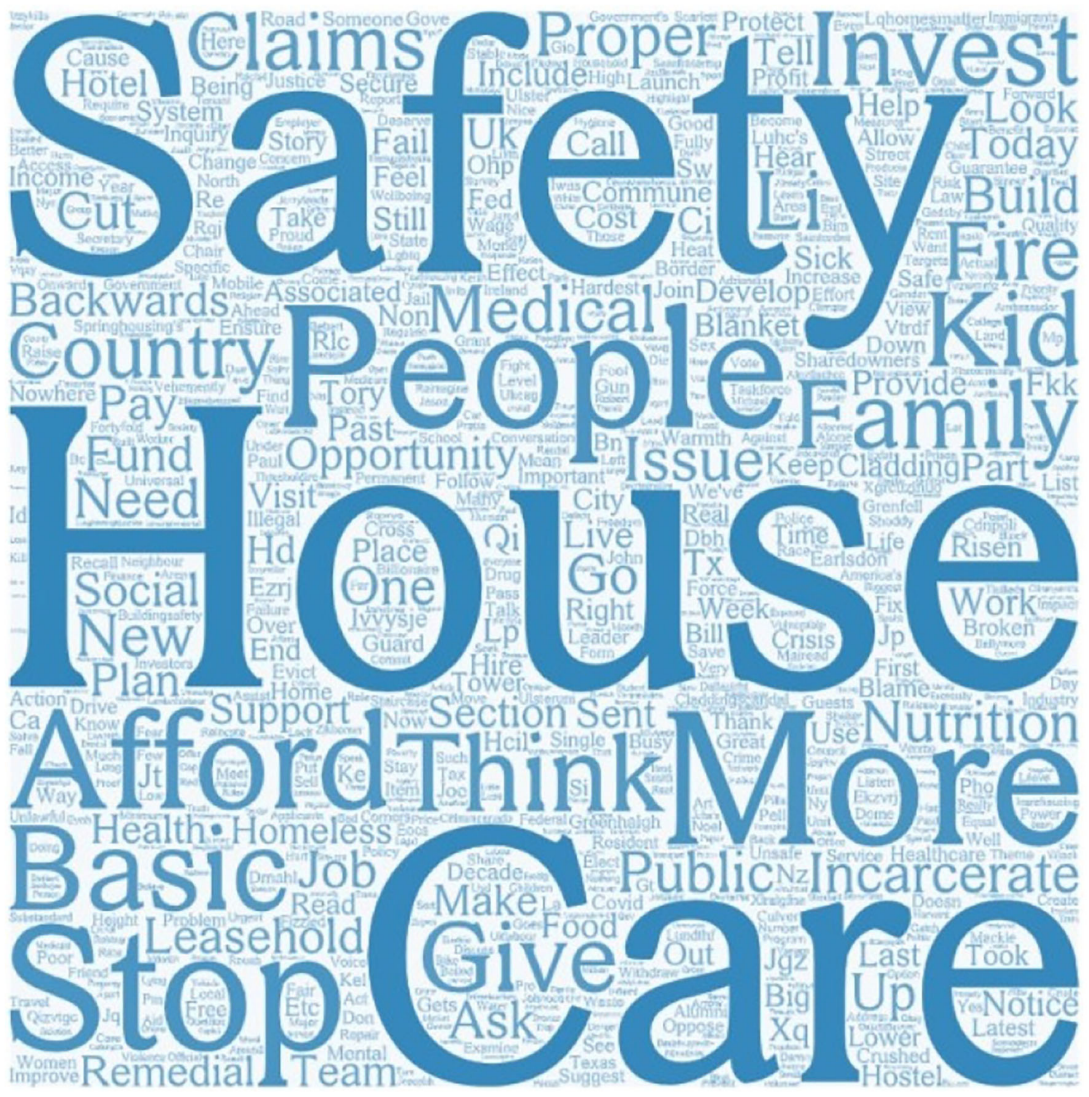
Most frequently mentioned words in housing safety.

We then performed sentiment analysis. Among 7,513 Tweets, 406 were strongly positive, 1,997 were positive, 4,087 were neutral, 978 were negative, and 45 were strongly negative. More than half of the Tweets were neutral; people did not talk about housing safety with strong emotions. In addition, word cloud identified some main concerns together with housing safety. “Afford,” “claims,” and “invest” refer to the fact that these people are concerned about finance together with safety. They also linked housing safety issues together with “family,” “kid,” and “people.” Then, “medical,” “fire,” and “nutrition” showed that they are aware of the potential threats to housing safety (Figure 9).

Residential safety had 685 Tweets. Among 685 Tweets, 38 were strongly positive, 267 were positive, 101 were neutral, 273 were negative, and six were strongly negative. According to in-degree centrality, the top 10 influencers are listed in the table, of which four opinion leaders are from Canada, and three are government departments. Most opinion leaders are professional users such as government, fire, counselors, and professors, apart from one opinion leader (veganowak, the only individual key opinion leader) who did not state his personal information. It also shows that the professional users are the primary group communicating residential safety issues on Twitter (Table 3).

**Table 3:** Key opinion leaders of residential safety in Twitter.

**Twitter ID**	**Description**	**Attribute**	**Place**
emergencyinfobc	We provide response and recovery info during provincial emergencies. Follow @PreparedBC for preparedness info. Collection Notice: https://t.co/u0vaxiXJjb	Government department	Canada
Preparedbc	Ready for a disaster? Get preparedness tips and recovery info here. Follow @EmergencyInfoBC for emergency information. Collection Notice: https://t.co/CSxYQ91LD9	Government department	Canada
Sffdpio	Official account for the SFFD. Serving CCSF/SFO/SF-PRESIDIO and GGNRA. For emergencies call or text 911; Non-life threatening hazards call 311. Not monitored 24/7	Fire department	US
ncc_ccn	Official feed for the National Capital Commission. Building a capital that inspires Canadians. FR: @CCN_NCC	Government organization	Canada
Willforster	Lib Dem Councilor for South Woking and Leader of the Lib Dems on Surrey County Council	Councilor	UK
Veganowak	No information	No information	No information
orla_hegarty	Architect, Asst Professor at @UCDDublin @UCDArch FRIAI RIBA ARB …opinions my own etc #Housing #Sustainability #Architecture #Construction #CovidIsAirborne	Professor	Ireland
Mathieufleury	#Ottawa rep. for #Lowertown #SandyHill #Vanier. #FrancoOntarien. Fan of #Sports & #politics. Care about #ottcity? Follow me!	Individual	Canada
Nfsaorg	National Fire Sprinkler Association	Organization	US
Mliebreich	CEO Liebreich Associates. Managing Partner EcoPragma Capital. Founder @BloombergNEF. UK Board of Trade. Chair @GoZeelo. Podcast: @MLCleaningUp. Olympic skibum.	Individual	UK

**Figure 10:**
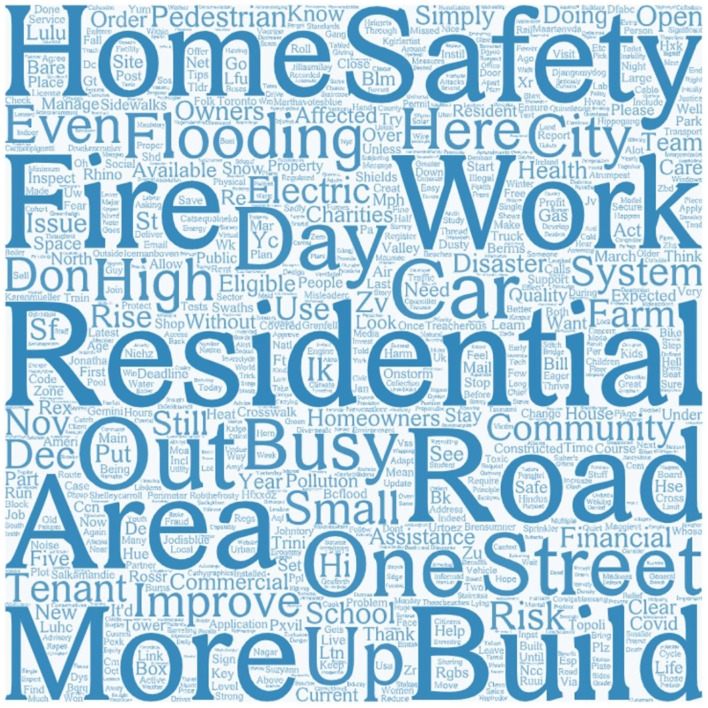
Most frequently mentioned words in residential safety Tweets.

According to the word cloud, the venue of residential safety Tweets centered on “home,” “road,” “street,” “city,” “farm,” and “community.” Second, “fire,” “car,” “flooding,” and “electric” showed frequent problems in residential safety (Figure 10). Finally, “owners” and “tenants” indicated that the stakeholders involved were house owners and tenants. There are 59 Tweets about housing health hazards. Among the top 10 key opinion leaders, six were from India, and four were government officials, which illustrates that Indians pay most attention to housing health hazard issues on Twitter. According to the in-degree centrality, the top 10 influencers are listed in the following table (Table 4).

**Table 4:** Key opinion leaders of residential safety Tweets.

**Twitter ID**	**Description**	**Attribute**	**Place**
Wsbgnl	Currently: data collector, occasional social scientist | recently: food delivery driver, retail clerk, food service worker, pet sitter, office assistant	Individual	US
Anexoplc	Anexo is a specialist integrated credit hire and legal services company acting for the Non-Fault Motorist. Listed on the AIM segment of the LSE (AIM: ANX)	Company	UK
Chalupaangry	Just like the planet, I'm on fire 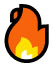	Individual	Unknown
Leadfreems	Lead Free Mississippi. Ending the threat of lead poisoning for Mississippi's children. Join by signing the pledge to Screen Birth to 6: https://t.co/QQp7H8UZfh	Organization	US
Pibmumbai	Zonal Office of Press Information Bureau @PIB_India, M/o Information & Broadcasting @MIB_India, Government of India, Mumbai, Maharashtra.	Government organization	India
iqbalsinghchah2	Commissioner of the Municipal Corporation of Greater Mumbai (BMC)	Government official	India
mayor_mumbai	Official account of the Mayor of Mumbai, Shrimati Kishori Pednekar	Government official	India
Authackeray	Voicing the Youth, Poems and Photography: Passion. President, Yuva Sena. President- Mumbai District Football Association Instagram: adityathackeray	Individual	India
aslamshaikh_mla	Cabinet Minister: Textiles, Port, Fisheries, and Guardian Minister-#Mumbai City. Constituency-Malad West (3 Term MLA), GS-MRCC. Government of #Maharashtra	Cabinet minister	India
cmomaharashtra	Office of the Chief Minister of Maharashtra	Government	India

**Figure 11:**
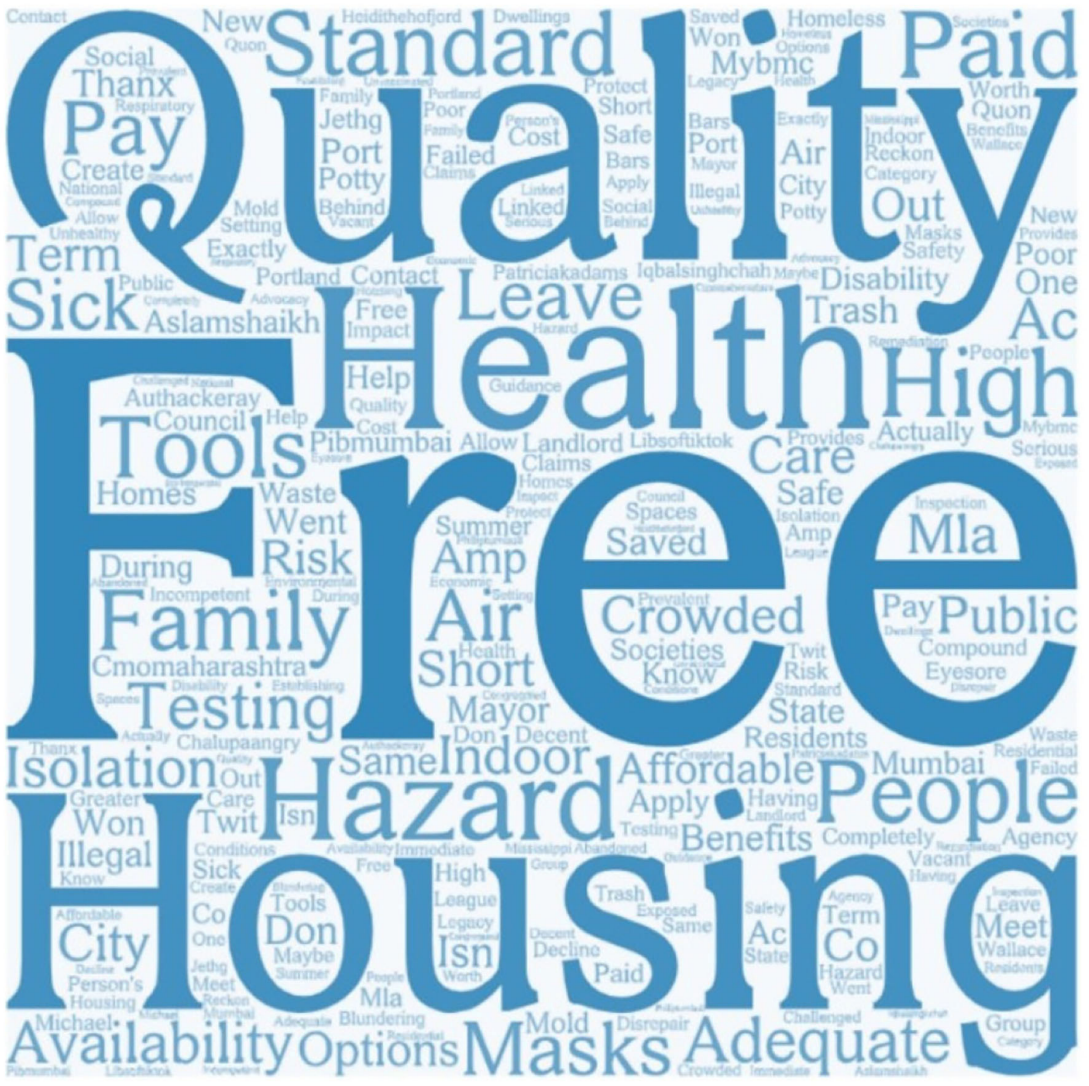
Most frequently mentioned words in housing hazard Tweets.

The sentiment analysis showed that out of 59 Tweets, 48 are positive, and 11 are negative. Word cloud keywords “free,” “paid,” “pay,” and “availability” indicated that people pay attention to the cost of reducing housing health hazards. “Family” and “public” were the most concerned about housing health hazard. “Air,” “mask,” “isolation,” “COVID,” and “isolation” showed that this issue is related to air quality and COVID. “Standard” indicated that people are concerned about the standards regarding the housing health hazard (Figure 11).

## Conclusion

Based on the bibliometric and visual analysis of housing safety research from 1945 to 2021, this study included 982 housing safety research articles. Since 1998, the research on housing safety and health has attracted the attention of more scholars, and the number of published articles has increased rapidly, accounting for 96% of all published articles. The United States and China have published more research articles than other countries. The United States has published the most significant number of publications, accounting for 30.76%. The number of published articles in China ranked second, accounting for 16.01% of published articles. The University of California in the United States and the University of London in the United Kingdom are the research institutions that issue the most articles. According to the statistics, 1,351 research institutions published articles in these areas, but 350 institutions jointly published two articles (≥2), accounting for 25.9% of all institutions. It shows that there is not much inter-institutional collaboration in this research area.

The research on housing safety was mainly published in Safety Science, American Journal of Public Health, International Journal of Environmental Research and Public Health, Habitat International, etc. There were 186 core authors in the housing safety study in our research. Analyzing published articles shows close cooperation within clusters and less cooperation outside clusters. It reflects that geographical proximity favors collaboration as fewer co-authors collaborate across regions and disciplines.

As the first systematically bibliometrics analysis on housing safety and health issues, our research innovatively uses AI sentimental analysis on all abstracts. The natural language processing algorithm results found that most research article abstracts about housing safety were positive. The number of positive articles was 521, accounting for 53.05% of the total. Nevertheless, there is a negative relationship between citations and emotions (−0.123), meaning abstracts with negative emotions attracted more citations. Despite many industries moving toward AI-related research, housing safety and health research are exceptions and have become a research void.

In the second part of the research, this study reviewed 8,257 Tweets. Most residential and housing Tweets came from Canada and the United Kingdom, respectively, while housing health hazards were from India. There were much more housing/residential safety (n = 8,198) Tweets than those in health (n = 59), which is the opposite to the academic research. The main concern about housing safety on Twitter includes finance, people, and threats to housing safety. Twitter users are mainly concerned about the cost of housing health issues, COVID, and air quality. Despite some applications like drones for crack detection *via* computer vision, AI robots and facial recognition have been used in housing estates, similar to academia, there is a lack of discussions linking AI to housing safety and health.

Top 10 housing safety influencers on Twitter worked for the government or were politicians. This shows that the government is concerned about housing safety. The top 10 opinion leaders regarding residential safety include the government departments, fire departments, counselors, and professors. Among them, four opinion leaders were Indian government officials. We may conclude that many government officers utilized Twitter as a channel to share housing/residential safety and health issues with the public. Individuals seldom use Twitter to express their opinions on these issues, in any case. The research results contribute to scholarship by comparing academic and public perceptions of housing safety and health. At the same time, the research methods provide a reference for researchers who try to use online social media, especially those who wish to use natural language methods, to process online data. Government officials might consider adopting Twitter data to solicit people's opinions, rather than surveys or interviews, which take a long time, and the response rate might be low (69).

## Data availability statement

The original contributions presented in the study are included in the article/supplementary material, further inquiries can be directed to the corresponding author/s.

## Author contributions

NL and RL performed the study, analyzed data, and wrote the manuscript. LS and QY contributed obtaining and analyzing data. RL and JD designed the study. NL and LS analyzed data and wrote the manuscript. All authors contributed to the article and approved the submitted version.

## Conflict of interest

The authors declare that the research was conducted in the absence of any commercial or financial relationships that could be construed as a potential conflict of interest.

## Publisher's note

All claims expressed in this article are solely those of the authors and do not necessarily represent those of their affiliated organizations, or those of the publisher, the editors and the reviewers. Any product that may be evaluated in this article, or claim that may be made by its manufacturer, is not guaranteed or endorsed by the publisher.

## References

[1] United Nations and the Rule of Law. The Human Right to Adequate Housing (Fact Sheet No. 21) (2009). Available online at: https://www.un.org/ruleoflaw/blog/document/the-human-right-to-adequate-housing-fact-sheet-no-21/ (accessed August 06, 2022).

[2] Li N Li RYM Pu R. What is in a name? A modern interpretation from housing price in Hong Kong. Pacific Rim Property Res J. (2021) 27:55–74. 10.1080/14445921.2021.1961182

[3] D'Alessandro D Appolloni L. Housing and health: an overview. Ann Ig. (2020) 32:17–26. 10.7416/ai.2020.3391 33146364

[4] Venable C Javernick-Will A Liel AB. Perceptions of post-disaster housing safety in future. Typhoons Earthq. (2020) 12:3837. 10.3390/su12093837

[5] Venable C Javernick-Will A Liel AB Koschmann MA. Revealing (mis)alignments between household perceptions and engineering assessments of post-disaster housing safety in typhoons. Int J Disast Risk Reduc. (2021) 53:101976. 10.1016/j.ijdrr.2020.101976

[6] Hirayama Y. Neoliberal policy and the housing safety net in Japan. City Cult Soc. (2010) 1:119–26. 10.1016/j.ccs.2010.10.001

[7] Arcury TA Weir MM Summers P Chen H Bailey M Wiggins MF. Safety, security, hygiene and privacy in migrant farmworker housing. (2012) 22:153–73. 10.2190/NS.22.2.d 22776578 PMC3710588

[8] Eterno JA. The public housing safety initiative in the Eastern District of New York: a collaborative researcher and practitioner program. In: Police Without Borders. Vol. 281-308. London: Routledge (2010). 10.1201/EBK1439805015-c12

[9] Ferreri M. Demunicipalisation, unaccountability by design and housing safety from below. Dialogues Human Geogr. (2021) 11:332–5. 10.1177/2043820620986404

[10] Bamzar R. Assessing the quality of the indoor environment of senior housing for a better mobility: a Swedish case study. J Housing Built Environ. (2019) 34:23–60. 10.1007/s10901-018-9623-4

[11] Fahimnia B Sarkis J Davarzani H. Green supply chain management: a review and bibliometric analysis. Int J Prod Econ. (2015) 162:101–14. 10.1016/j.ijpe.2015.01.003

[12] Chen WY. Environmental externalities of urban river pollution and restoration: a hedonic analysis in Guangzhou (China). Urban Plan. (2017) 157:170–9. 10.1016/j.landurbplan.2016.06.010

[13] Ban J Chen L. Evaluation of the factors influencing the housing safety awareness of residents in Shanghai. PLoS ONE. (2020) 15:e0227871. 10.1371/journal.pone.0227871 31978070 PMC6980500

[14] Husin HN Nawawi AH Ismail F Khalil N. Correlation analysis of occupants' satisfaction and safety performance level in low-cost housing. In: Asia Pacific International Conference on Environment-Behaviour Studies (AICE-BS 2014 Berlin), Vol. 168. (2015). p. 238–48. 10.1016/j.sbspro.2014.10.229

[15] El-Anwar O El-Rayes K Elnashai A. Maximising temporary housing safety after natural disasters. J Infrastruct Syst. (2010) 16:138–48. 10.1061/(ASCE)IS.1943-555X.0000018

[16] Hui L. Study on safety management of the temporary community after the earthquake. In: International Symposium on Safety and Engineering in China. (2012). p. 214–20. 10.1016/j.proeng.2012.08.037

[17] Hassanain MA Hafeez MA Sanni-Anibire MO. A ranking system for fire safety performance of student housing facilities. Saf Sci. (2017) 92:116–27. 10.1016/j.ssci.2016.10.002

[18] Dzolev I Laban M Draganic S. Survey based fire load assessment and impact analysis of fire load increment on fire development in contemporary dwellings. Saf Sci. (2021) 135:105094. 10.1016/j.ssci.2020.105094

[19] El-Anwar O El-Rayes K Elnashai A. An automated system for optimizing post-disaster temporary housing allocation. Autom Construct. (2009) 18:983–93. 10.1016/j.autcon.2009.05.003

[20] Mo X Wang W. Review on a practical approach of sustainable urban design strategy in the perspective of conflict in Shanghai. Int Rev Spatial Plan Sustain Dev. (2014) 2:44–53. 10.14246/irspsd.2.4_44

[21] Mao Y Huang H Liu Y. Research on government supervision system of housing safety appraisal institution. In: 2017 7th International Conference on Education and Management (ICEM 2017). Atlantis Press (2018). 10.2991/icem-17.2018.160

[22] Yu D Fang C. The dynamics of public safety in cities: a case study of Shanghai from 2010 to 2025. Habitat Int. (2017) 69:104–13. 10.1016/j.habitatint.2017.09.007

[23] Chan FKS Griffiths JA Higgitt D Xu S Zhu F Tang YT. “Sponge City” in China—a break through of planning and flood risk management in the urban context. Land Use Policy. (2018) 76:772–8. 10.1016/j.landusepol.2018.03.005

[24] Bhusal JK. Citizen science for hydrological risk reduction and resilience building. Wiley Interdiscip Rev Water. (2018) 5:e1262. 10.1002/wat2.1262

[25] Qian Q Lin P. Safety risk management of underground engineering in China: progress, challenges and strategies. J Rock Mech Geotech Eng. (2016) 8:423–42. 10.1016/j.jrmge.2016.04.001

[26] Leung KW Yau JH Roberds W. Challenges in applying landslide risk management to housing developments in Hong Kong. Landslide Risk Assess.(2018) 251–9. 10.1201/9780203749524-15

[27] Shan L Ann TW Wu Y. Strategies for risk management in urban-rural conflict: Two case studies of land acquisition in urbanizing China. Habitat Int. (2017) 59:90–100. 10.1016/j.habitatint.2016.11.009 32287707 PMC7124285

[28] Yu T Shen GQ Shi Q Lai X Li CZ Xu K. Managing social risks at the housing demolition stage of urban redevelopment projects: a stakeholder-oriented study using social network analysis. Int J Project Manag. (2017) 35:925–41. 10.1016/j.ijproman.2017.04.004

[29] Li CZ Hong J Xue F Shen GQ Xu X Luo L. SWOT analysis and Internet of Things-enabled plat form for prefabrication housing production in Hong Kong. Habitat Int. (2016) 57:74–87. 10.1016/j.habitatint.2016.07.002

[30] Braubach M. Key challenges of housing and health from WHO perspective. Int J Public Health. (2011) 56:579–80. 10.1007/s00038-011-0296-y 21894569

[31] DunnJRHayesMVHulchanskiJDHwangSWPotvinL. Housing as a socio-economic determinant of health: findings of a national needs, gaps and opportunities assessment. Can J Public Health. (2006) 97:S12–7. 10.1007/BF0340539217357542

[32] Easterlow D Smith SJ. Housing for health: can the market care? Environ Plan A Econ Space. (2004) 36:999–1017. 10.1068/a36178

[33] Baker E Beer A Lester L Pevalin D Whitehead C Bentley R. Is housing a health insult? Int J Res Public Health. (2017) 14:567. 10.3390/ijerph14060567 28587139 PMC5486253

[34] Arku G Luginaah I Mkandawire P Baiden P Asiedu AB. Housing and health in three contrasting neighbourhoods in Accra, Ghana. Soc Sci Med. (2011) 72:1864–72. 10.1016/j.socscimed.2011.03.023 21561698

[35] Gibson M Petticrew M Bambra C Sowden AJ Wright KE Whitehead M. Housing and health inequalities: a synthesis of systematic reviews of interventions aimed at different pathways linking housing and health. Health Place. (2011) 17:175–84. 10.1016/j.healthplace.2010.09.011 21159542 PMC3098470

[36] Lawrence RJ. Housing and health: from interdisciplinary principles to transdisciplinary research and practice. Futures. (2004) 36:487–502. 10.1016/j.futures.2003.10.001

[37] Lawrence RJ. Housing and health: beyond disciplinary confinement. J Urban Health. (2006) 83:540–9. 10.1007/s11524-006-9055-4 16739053 PMC2527187

[38] Ziersch A Walsh M Due C Duivesteyn E. Exploring the relationship between housing and health for refugees and asylum seekers in South Australia: a qualitative study. Int J Environ Res Public Health. (2017) 14:1036. 10.3390/ijerph14091036 28885594 PMC5615573

[39] Yin Z Sulieman LM Malin B. A systematic literature review of machine learning in online personal health data. J Am Med Inform Assoc. (2019) 26:561–76. 10.1093/jamia/ocz009 30908576 PMC7647332

[40] Yao Q Li RYM Song L Crabbe MJC. Construction safety knowledge sharing on Twitter: a social network analysis. Saf Sci. (2021) 143:105411. 10.1016/j.ssci.2021.105411

[41] Liu Y Yan C Yin Z Wan Z Xia W Kantarcioglu M. Biomedical research cohort membership disclosure on social media. In: AMIA Annual Symposium Proceedings. (2019). p. 607–16. 32308855 PMC7153128

[42] Ineichen B. Homes and Health: How Housing and Health Interact. London: Routledge (2003). 10.4324/9780203473900

[43] Song L Li RYM Yao Q. An informal institution comparative study of occupational safety knowledge sharing via French and English Tweets: languaculture, weak-strong ties and AI sentiment perspectives. Saf Sci. (2022) 147:105602. 10.1016/j.ssci.2021.105602

[44] Sever M Ozdemir S Jobson K. ‘An academic is like a bad dinner guest.' Exploring cross-cultural perspectives of academics via metaphors. High Educ Res Dev. (2022) 14:1262–76. 10.1080/07294360.2021.1887096

[45] Shapin S. The ivory tower: the history of a figure of speech and its cultural uses. Br J History Sci. (2012) 45:1–27. 10.1017/S0007087412000118

[46] Ni C Wan Z Yan C Liu Y Clayton E Malin B Yin Z. The Public perception of the #GeneEditedBabies event across multiple social media platforms: observational study. J Med Internet Res. (2022) 24:e31687. 10.2196/31687 35275077 PMC8957000

[47] Davydenko M Kolbuszewska M Peetz J. A meta-analysis of financial self-control strategies: comparing empirical findings with online media and lay person perspectives on what helps individuals curb spending and start saving. PLOS ONE. (2021) 16:e0253938. 10.1371/journal.pone.0253938 34237109 PMC8266115

[48] Fetscherin M Heinrich D. Consumer brand relationships research: a bibliometric citation meta-analysis. J Bus Res. (2015) 68:380–90. 10.1016/j.jbusres.2014.06.010

[49] Nerur SP Rasheed AA Natarajan V. The intellectual structure of the strategic management field: an author co-citation analysis. Strat Manag J. (2008) 29:319–36. 10.1002/smj.659

[50] Chen Y Liu Z. Quietly rising map of scientific knowledge. Sci Res. (2005) 2:149–54.

[51] Rodriguez A Laio A. Clustering by fast search and find of density peaks. Science. (2014) 344:1492–6. 10.1126/science.1242072 24970081

[52] Zupic I Cater T. Bibliometric methods in management and organization. Organ Res Method. (2015) 18:429–72. 10.1177/1094428114562629

[53] Zhou W Xu Z Zavadskas EK. A bibliometric overview of the International Journal of Strategic Property Management between 2008 and 2019. Int J Strat Property Manag. (2019) 23:366–77. 10.3846/ijspm.2019.10535

[54] Moghayedi A Awuzie B Omotayo T Le Jeune K Massyn M Ekpo CO. A critical success factor framework for implementing sustainable innovative and affordable housing: a systematic review and bibliometric analysis. Buildings. (2021) 11:317. 10.3390/buildings11080317

[55] Ramos-Rodriguez A Ruiz-Navarro J. Changes in the intellectual structure of strategic management research: a bibliometric study of the Strategic Management Journal, 1980–2000. Strategic Manag J. (2004) 25:981–1004. 10.1002/smj.397

[56] Yin X Liu H Chen Y Al-Hussein M. Building information modelling for offsite construction: review and future directions. Autom Construct. (2019) 101:72–91. 10.1016/j.autcon.2019.01.010

[57] Daim TU Rueda G Martin H Gerdsri P. Forecasting emerging technologies: use of bibliometrics and patent analysis. Technol Forecast Social Change. (2006) 73:981–1012. 10.1016/j.techfore.2006.04.00433594568

[58] Xia W Wan Z Yin Z Gaupp J Liu Y Clayton EW. It's all in the timing: calibrating temporal penalties for biomedical data sharing. J Am Med Inform Assoc. (2018) 25:25–31. 10.1093/jamia/ocx101 29036325 PMC6080807

[59] Van Eck NJ Waltman L. Software survey: VOSviewer, a computer program for bibliometric mapping. Scientometrics. (2010) 84:523–38. 10.1007/s11192-009-0146-3 20585380 PMC2883932

[60] Song X Chi P. Comparative study on the application of VOSviewer and Citespace. Inform Sci. (2016) 34:108–12. 10.13833/j.cnki.is.2016.07.021

[61] Ye C. Knowledge map of international urban agriculture research-quantitative analysis based on Citespace and VOSviewer. Chin Agric Sci Bull. (2019) 35:134–43. 10.11924/j.issn.1000-6850.casb17090127

[62] Van Eck NJ Waltman L. VOSviewer Manual. (2020). Available online at: https://www.vosviewer.com/ (accessed August 06, 2022).

[63] Tang LY Xiong C Wang Y Zhou YB Zhao ZJ. Review of Deep Learning for Short Text Sentiment Tendency Analysis. J Front Comput Sci Technol. (2021) 15:079418. 10.3778/j.issn.1673-9418.2010002

[64] Zulkifli NSA Lee AWK. Sentiment analysis in social media based on english language multilingual processing using three different analysis techniques. In: International Conference on Soft Computing in Data Science. Berlin: Springer (2019). p. 375–85.

[65] Moher D Liberati A Tetzlaff J Altman DG Group P. (2009). Preferred reporting items for systematic reviews and meta-analyses: the PRISMA statement. PLoS Med. 6:97. 10.1371/journal.pmed.1000097 21603045 PMC3090117

[66] Hallinger P Chatpinyakoop C. A bibliometric review of research on higher education for sustainable development, 1998–2018. Sustainability. (2019) 11:2401. 10.3390/su11082401

[67] Cao M Li Y Gou YX. Knowledge mapping analysis of antibiotics in soil research based on the CiteSpace software. J Agric Resour Environ. (2020) 37:627–35. 10.13254/j.jare.2019.0396

[68] Goodwin MB Fontenla M Gonzalez F. Estimating the impact of pollution on wages and housing prices using satellite imagery. Appl Econ Lett. (2020) 28:1750–3. 10.1080/13504851.2020.1853665

[69] Li RYM Chau KW Ho DCW. Current State of Art in Artificial Intelligence and Ubiquitous Cities. Singapore: Springer (2022).

